# Vitamin D levels in children with familial Mediterranean fever

**DOI:** 10.1186/s12969-016-0089-1

**Published:** 2016-04-27

**Authors:** Hatice Onur, Hale Aral, Vefik Arica, Gamze Atalay Bercem, Ozgur Kasapcopur

**Affiliations:** Department of Pediatrics, Bursa Yuksek Ihtisas Training and Research Hospital, 152 Evler, Prof. Tezok street No: 2, Yıldırım/Bursa, Istanbul, Turkey; Department of Biochemistry, Istanbul Training and Research Hospital, Istanbul, Turkey; Department of Pediatrics, Yeniyuzyıl University, Gaziosmanpasa Hospital, Istanbul, Turkey; Department of Pediatrics Rheumatology, Cerrahpasa Medical Faculty, Istanbul University, Istanbul, Turkey

**Keywords:** 25-hydroxyvitamin D, Familial Mediterranean fever, Children, Gene mutation

## Abstract

**Background:**

This study aimed to determine whether vitamin D deficiency is more common in children with familial Mediterranean fever (FMF) than in healthy individuals.

**Methods:**

The study group consisted of 100 patients diagnosed with FMF and 50 healthy children. Serum baseline 25-hydroxyvitamin D levels and other related parameters were evaluated.

**Results:**

The mean (standard deviation [SD]) vitamin D levels in patients with FMF and healthy controls were 24.78 (8.35) and 28.70 (11.70) ng/mL, respectively. Patients with FMF had significantly decreased vitamin D levels compared with those in healthy controls (*P* = 0.039). Vitamin D levels were similar in patients with FMF with different *MEFV* mutations (*P* = 0.633). Age was significantly correlated with vitamin D levels (*r* = −0.235, *P* = 0.019). In addition, a negative correlation between parathyroid hormone and vitamin D levels was detected (r_s_ = −0.382, *P* < 0.0001).

**Conclusion:**

This study demonstrated that vitamin D levels are lower in children with FMF than in healthy controls. We speculate that vitamin D levels should be carefully examined, and nutritional supplementation may be required in patients with FMF. Further studies with larger patient populations are needed to confirm the frequency of vitamin D deficiency in patients with FMF.

## Background

Familial Mediterranean fever (FMF) is an autosomal recessive disease that is prevalent among eastern Mediterranean populations, mainly non-Ashkenazi Jews, Armenians, Turks, and Arabs [1]. Patients experience recurrent, self-limiting inflammatory febrile attacks, as well as abdominal, chest, and joint pain. The factors that trigger or terminate these periodic attacks are unknown. Some patients with FMF have chronic immune activation, reflected by subtle clinical signs of inflammation, such as chronic normocytic normochromic anemia, splenomegaly, decreased bone mineral density, persistent elevation of fibrinogen levels and erythrocyte sedimentation rates (ESRs), and growth retardation [2].

In recent years, the discovery of vitamin D receptors on immune cells and the fact that several of these cells produce vitamin D suggest that vitamin D may have immunoregulatory properties. Vitamin D, in addition to phosphorus and calcium, has pleiotropic immunomodulating effects [3]. Vitamin D status has been linked to the occurrence and severity of autoimmune and inflammatory diseases [4].

FMF is an inflammatory disease that is more common in populations surrounding Mediterranean sea. The disease is prevalent in 0.1 % of the Turkish population, and one in five individuals is a carrier of mutations [1]. Our study is valuable, as it includes a large number of pediatric patients with FMF. As low-grade inflammation occurs in FMF, we aimed to determine whether vitamin D deficiency is more prevalent in children with FMF than in healthy individuals.

## Methods

### Subjects

The study group included 100 patients with a diagnosis of FMF who received medical care at Istanbul Cerrahpasa Medical Faculty of Rheumatology outpatient clinics. Patients with other known chronic diseases were excluded. None of the patients was in the attack period of FMF. All patients were receiving treatment with colchicine, whereas no patient in the control group was receiving any medication that could affect vitamin D levels. Blood samples were drawn in the summer (June-July 2013). The control group comprised 50 age-and sex-matched healthy children. Blood samples were taken from the healthy controls for another medical reason with parent approval. The diagnosis of FMF was established according to criteria defined by Yalcınkaya et al. [5]. The study was approved by the local ethics committee of the Istanbul Education and Research Hospital and was conducted in accordance with the Declaration of Helsinki.

### Measurements

Blood samples were obtained from patients with FMF and controls. Serum samples for vitamin D measurements were obtained by centrifugation within 2 h after blood samples were drawn and frozen at −80 °C prior to analysis. The frozen samples were transferred properly (on dry ice in the dark) to Acıbadem Labmed (Istanbul, Turkey). The experiments were performed using an Agilent 6460 triple quadrupole liquid chromatography (LC)/mass spectrometry system equipped with an electrospray ionization ion source interface in the positive ion mode (Agilent Technologies, Santa Clara, CA, USA). The lower detection limit was 3.0 ng/mL. At a mean of 16.13 ng/mL, the inter-assay coefficient value (CV) was 3.37 %, and at a mean of 50.96 ng/mL, the inter-assay CV was 1.52 % (Chromsystems Mass Check, Gräfelfing, Germany).

In the Istanbul Education and Research Hospital Central Biochemistry Laboratory, routine biochemical analyses of serum samples were performed using an Advia 2400 Chemistry System. Parathyroid hormone (PTH) levels were measured using a Centaur XP hormone analyzer, fibrinogen content was determined using a Sysmex CA-1500 automated coagulometer, and C-reactive protein (CRP) levels were measured using a BNII nephelometer (Siemens Healthcare Diagnostics Inc).

Gene mutations were detected previously and included in the retrospective file records of the patients with FMF. Plasma vitamin D levels were categorized as follows: sufficient, 25–80 ng/mL; mild-moderate deficiency, 10–24 ng/mL; and severe deficiency, 0–10 ng/mL. These reference intervals for serum 25. OH-D vit levels were reported by Acıbadem Labmed (Istanbul, Turkey) compatible with the literature [6]. The reference intervals for vitamin D metabolites are method-dependent, and the lower limit of 25.OH-D vit levels considered sufficient for health is controversial, as vitamin D levels vary according to season, localization, skin type, nutritional status, sun exposure, and lifestyle [7].

### Statistical analyses

Statistical analysis was performed using the Number Cruncher Statistical System 2004 (NCSS Systems, Kaysville, UT, USA) and MedCalc (MedCalc Software, Broekstraat, Mariakerke, Belgium). Kolmogorov-Smirnov goodness-of-fit tests were used to determine whether the variables exhibited a Gaussian distribution. Normally distributed numerical variables were expressed as the mean (standard deviation [SD]) if normally distributed, and values that lacked a normal distribution were expressed as the median (interquartile range [IQR]). Pearson’s chi-squared tests were used to compare categorical variables. Statistical comparisons of the two groups were performed using Student’s *t*-tests for data with a Gaussian distribution or Mann–Whitney U-tests for data with a non-Gaussian distribution. Pearson’s correlation coefficient (r), Spearman’s rank correlation coefficient (r_s_), or a biserial correlation coefficient was used to evaluate the degree of association between two variables. Two-way analysis of variance was performed to identify a significant interaction effect between the independent variables. All statistical tests were two-sided, and *P* < 0.05 denoted statistical significance.

## Results

The demographic characteristics and main laboratory parameters of the patients and controls are summarized in Table 1. Among the patients with FMF, the median age at diagnosis of FMF was 6 years (IQR, 4–10 years), and the median duration since onset was 3 years (IQR, 2–5 years). In total, 48 (48 %) patients had articular findings, and no patients had renal involvement. The presenting symptoms were abdominal pain and fever for 39 (39 %) patients, abdominal pain/fever/arthritis/arthralgia for 28 (28 %) patients, isolated arthritis/arthralgia for 8 (8 %) patients, abdominal pain/fever/skin lesions for 5 (5 %) patients, fever/arthritis/arthralgia for 5 (5 %) patients, and other symptoms (chest pain, myalgia, and skin lesions) for 13 (13 %) patients. Additionally, 37 (37 %) patients had a family history of FMF.

**Table 1:** Demographic characteristics and main laboratory parameters of patients with and without familial Mediterranean fever

	Familial Mediterranean fever group (mean [SD]) (*n* = 100)	Control group (mean [SD]) (*n* = 50)	*P*-value
Age at investigation (years)	10.8 (3.5)^a^	10.4 (4.4)^a^	0.560
Female/male (*n*)	54/46	25/25	0.644
Age at diagnosis (years)	6 (4–10)^b^		
Duration of colchicine use (years)	3 (2–5)^b^		
Fibrinogen (mg/dL)	*288 (63)* ^b^		
CRP (mg/dL)	0.20 (0.11–0.41)^b^		
ESR (mm/h)	13 (9–21)^b^		
Alkaline phosphatase (U/L)	215 (80)^a^		
*MEFV* mutation test results (*n*)			
M694V/M694V	16		
M694V/other	34		
Other/other	37		
Negative	13		

*SD* standard deviation, *CRP* C-reactive protein, *ESR* erythrocyte sedimentation rate

Mean(SD)^a^

Median(IQR)^b^

Serum levels of calcium, phosphorus, PTH, and vitamin D were compared between the groups (Table 2). The mean (SD) vitamin D levels in patients with FMF and healthy controls were 24.78 (8.35) and 28.70 (11.70) ng/mL, respectively. Plasma vitamin D levels were significantly lower in patients with FMF than in the controls (*P* = 0.039)

**Table 2:** Serum calcium, phosphorus, PTH, and plasma vitamin D levels in patients with and without familial Mediterranean fever

	Familial Mediterranean fever group (*n* = 100)	Control group (*n* = 50)	*P*-value
Calcium (mg/dl) (mean [SD])	9.6 (0.5)	9.8 (0.4)	<0.01
Phosphorus (mg/dl) (median [IQR])	4.9 (4.5–5.2)	4.8 (4.4–5.1)	0.318
PTH (pg/mL) (median [IQR])	49.9 (28.7–64.8)	34.2 (24.2–53.2)	0.024
25-OH vitamin D (ng/mL) (mean [SD])	24.78 (8.35)	28.70 (11.70)	0.039

*PTH* parathyroid hormone, *SD* standard deviation, *IQR* interquartile range

Vitamin D levels were deficient in 38.0 % (19/50) and sufficient levels in 62.0 % (31/50) of control group. In addition 57.0 % (57/100) were deficient and 43.0 % (43/100) were in sufficient levels of FMF patients. (*P* = 0.024) (Table 3). Pearson’s chi-square test was used to compare observed levels with expected frequencies. The deficiency of the patients according to frequency of mild,moderate and severe deficiencies were %2 (2/100), %31 (31/100), %26 (26/100) and for the control group %6 (3/50), %14 (7/50),%16 (8/50) respectively.

**Table 3:** Vitamin D levels according to cut off levels

	Familial Mediterranean fever group (*n* = 100)	Control group (*n* = 50)	*P*-value
Vitamin D < 25 ng/mL (n, %)	57 (57.0 %)	19 (38.0 %)	0.024
Vitamin D = 25–80 ng/mL (n, %)	43 (43.0 %)	31 (62.0 %)	

An increase in age was significantly correlated with a decrease in vitamin D levels (*r* = −0.235, *P* = 0.019). Additionally, a negative correlation was detected between PTH and vitamin D levels (r_s_ = −0.382, *P* < 0.0001). No significant correlation was noted between vitamin D levels and the ESR or CRP levels.

Plasma vitamin D levels were not significantly different between patients with (*n* = 48) and without articular symptoms (*n* = 52) (23.72 [7.93] ng/mL versus 25.20 [8.99] ng/mL, *P* = 0.328).

Meanwhile, plasma vitamin D levels were similar among patients with FMF with different *MEFV* mutations (*P* > 0.05) (Table 4). The most prevalent mutations detected as *M694V HT* (*N* = 18,%18) and *M694 HM* (*N* = 16,%16) respectively.

**Table 4:** Vitamin D levels in patients with familial Mediterranean fever and different *MEFV* mutations

	M694V/M694V (*n* = 16)	M694V/Other (*n* = 34)	Other/Other (*n* = 37)	Negative (*n* = 13)	*P*-value
Vitamin D (ng/mL) (mean [SD])	23.71 (9.94)	26.35 (9.84)	23.85 (5.52)	24.64 (9.07)	0.633

*SD* standard deviation

## Discussion

Our study established that vitamin D deficiency is present in a large number of Turkish children diagnosed with FMF.

The influence of vitamin D deficiency on inflammation is being explored, but studies have not demonstrated a causative effect. FMF is autoinflammatory disease in which *MEFV* mutations change pyrin protein and thus inflammasome activity in cells of innate immune system [2]. The vitamin D effect on innate immun cells also investigated in other autoinflamatuar diseases like Behcet Disease(B.D.).Do JE et al. underlined the immunomodulatrory effect of vitamin D through down regulation of Toll-like receptor(TLR) expression in human monocytes. They also suggested that vitamin D may have an immunomodulatory effect on innate immunity-mediated inflammation in BD [8].

Some researchers hypothesized that low vitamin D is the consequence of a chronic inflammatory process caused by persistent infection [9]. The mechanism by which vitamin D reduces inflammation remains poorly understood. T helper cells playing a crucial role in FMF pathogenesis produce interferon (IFN)-gamma and tumor necrosis factor (TNF)-beta [10, 11]. Aypar et al. identified elevated IFN-gamma levels produced by Th-1 cells during FMF attacks compared to those in healthy controls and suggested that Th-1 polarization may trigger inflammation in patients with FMF [12]. Vitamin D, which possesses immunomodulatory effects, inhibits the production of interleukin (IL)-6 and IFN-gamma by reducing the differentiation and maturation of myeloid, Th-1, and Th-17 cells [13, 14]. Koklu et al. also reported elevated IFN gamma levels in patients with FMF during both attack and attack-free periods [15]. Additionally, some researchers connected low vitamin D levels to malabsorption of the vitamin as a result of colchicine treatment. Padeh et al. reported that 14 % of patients developed diarrhea during colchicine treatment [16].

We detected significantly lower serum 25-hydroxyvitamin D levels among patients with FMF than in matched controls (*P* < 0.01), and female patients with FMF were most strongly affected. Female (*N* = 54) and male (*N* = 46) patient’s mean vitamin D levels were 23.09 (18.79–26.53) and 26.01 (20.79–32.35) respectively (*P* = 0.014). Most previous studies were conducted in adults with FMF. Recently, Erten et al. [17] and Kisacik et al. [18] detected significantly lower serum 25-hydroxyvitamin D levels among patients with FMF than in matched controls (*P* < 0.001). Female patients with FMF were also most greatly affected in the study by Erten et al. [17]. They associated this result with styles of dress, low activity levels, and inadequate exposure to sunlight.

Chronic inflammation is associated with a decrease in bone mineral density in patients with various inflammatory diseases, and attack-free patients with FMF have considerably lower T-scores at the femur neck and lumbar spine than healthy individuals, reflecting their increased risk of developing early-onset osteoporosis [19–21]. Inflammatory activity in patients with FMF plays a major role in the pathophysiology of bone loss. This may be mediated by substances regulating both the inflammatory process and bone turnover [22]. Alterations in vitamin D metabolism can cause increases in bone resorption [23]. Osteoporosis in patients with FMF can also be related to decreased vitamin D levels [17]. Similarly, we detected a negative correlation between vitamin D and PTH levels, and a negative correlation between alkaline phosphatase and vitamin D levels has been reported, emphasizing the effects of vitamin D deficiency in patients with FMF. We propose that convenient nutritional supplementation would prevent the likely development of osteoporosis in patients with FMF. In a recent study, Zhang et al. supported the idea that serum vitamin D levels should be maintained at more than 30 ng/mL in the physiologic range to achieve sufficient anti-inflammatory effects [24]. Erten et al. concluded that vitamin D levels were significantly lower in adult patients who presented with joint symptoms as the first presentation than in those who presented with abdominal attacks. In our study, plasma vitamin D levels were not significantly different between patients with and without articular symptoms [17]. We speculate that this finding may be associated with the ages of patients in our study.

Some authors also detected increased inflammation in carriers of *MEFV* mutations [25]. We investigated this association, but no significant correlation was detected (*P* = 0.633). Erten et al. [17] demonstrated that increased levels of inflammatory markers (ESR and fibrinogen) in patients with FMF were correlated with lower vitamin D levels. We also investigated this association but found no significant correlations among these markers (*P* > 0.05). This indicates that colchicine treatment suppressed inflammation. Increased inflammation exists in the attack period, and thus, we are planning to investigate vitamin D levels in this period in our future studies.

In a recent study, Anık et al. [26] also detected decreased vitamin D levels in children and indicated that the cumulative colchicine dose appears to negatively affect vitamin D levels. It was concluded that colchicine inhibited the functions of intracellular microtubules, which are essential components of the normal genomic response to vitamin D. Defects in the microtubular network attributable to colchicine use may reduce vitamin D levels because of increased production of 1,25(OH) vitamin D and 24,25(OH) vitamin D [26, 27]. Future studies should investigate the potential association between the colchicine dose and vitamin D status.

Our study had some limitations. We could not consider variables that can influence vitamin D levels such as dietary intake, physical activity status, sunlight exposure, increased skin pigmentation, and genetic polymorphisms of vitamin D receptors. In addition, the associations among the cumulative colchicine dose, acute attack periods, and severity scores have not been examined thoroughly. Our planned studies will investigate these associations.

To date, few studies have investigated vitamin D deficiency in patients with FMF. As our study is the first to include a large number of patients with FMF and controls, it has significant validity.

## Conclusion

In conclusion, scientific research has not clarified whether vitamin D deficiency is a consequence or cause of inflammatory disease. This study demonstrated that vitamin D levels are lower in children with FMF than in healthy controls. We speculate that vitamin D levels should be carefully examined, and nutritional supplementation may be required in patients with FMF. Further studies with larger patient populations are needed to investigate vitamin D deficiency in patients with FMF.

## Abbreviations

CRP: C-reactive protein
ESR: erythrocyte sedimentation rate
FMF: familial Mediterranean fever
IFN: interferon
MEFV: mediterranean fever
PTH: parathyroid hormone
SD: standard deviation
Th-1: T helper-1
TLR: toll-like receptor
TNF: tumor necrosis factor
